# Modifications to the National Early Warning Score 2: a Scoping Review

**DOI:** 10.1186/s12916-025-03943-0

**Published:** 2025-03-11

**Authors:** Victoria Riccalton, Lynsey Threlfall, Ananya Ananthakrishnan, Cen Cong, Madison Milne-Ives, Peta Le Roux, Chris Plummer, Edward Meinert

**Affiliations:** 1 Translational and Clinical Research Institute, Newcastle University, Newcastle Upon Tyne, NE4 5PL UK; 2 Newcastle Upon Tyne Hospitals NHS Foundation Trust, Newcastle Upon Tyne, NE7 7DN UK; 3 Centre for Health Technology, School of Nursing and Midwifery, University of Plymouth, Plymouth, PL4 8AA UK; 4 NIHR Newcastle Biomedical Research Centre, Newcastle University, Newcastle Upon Tyne, NE1 7RU UK; 5 Department of Primary Care and Public Health, School of Public Health, Imperial College London, London, W6 8RP UK

**Keywords:** Early warning score, National early warning score, NEWS2, Accuracy, Sensitivity and specificity, Clinical decision-making, Risk management

## Abstract

**Background:**

The National Early Warning Score 2 (NEWS2) has been adopted as the standard approach for early detection of deterioration in clinical settings in the UK, and is also used in many non-UK settings. Limitations have been identified, including a reliance on ‘normal’ physiological parameters without accounting for individual variation.

**Objective:**

This review aimed to map how the NEWS2 has been modified to improve its predictive accuracy while placing minimal additional burden on clinical teams.

**Methods:**

The Preferred Reporting Items for Systematic Reviews and Meta-Analyses (PRISMA-ScR) and the Population, Intervention, Comparator, Outcome, and Study (PICOS) frameworks were followed to structure the review. Six databases (CINAHL, PubMed, Embase, ScienceDirect, Cochrane Library and Web of Science) were searched for studies which reported the predictive accuracy of a modified version of NEWS2. The references were screened based on keywords using EndNote 21. Title, abstract and full-text screening were performed by 2 reviewers independently in Rayyan. Data was extracted into a pre-established form and synthesised in a descriptive analysis.

**Results:**

Twelve studies were included from 12,867 references. In 11 cases, modified versions of NEWS2 demonstrated higher predictive accuracy for at least one outcome. Modifications that incorporated demographic variables, trend data and adjustments to the weighting of the score’s components were found to be particularly conducive to enhancing the predictive accuracy of NEWS2.

**Conclusions:**

Three key modifications to NEWS2—incorporating age, nuanced treatment of FiO_2_ data and trend analysis—have the potential to improve predictive accuracy without adding to clinician burden. Future research should validate these modifications and explore their composite impact to enable substantial improvements to the performance of NEWS2.

## Background

The National Early Warning Score (NEWS) was developed to improve and standardise the performance of the UK National Health Service (NHS) in the detection of acute illness and clinical deterioration. It was introduced as a standardised national approach to the assessment of, and response to, patients presenting with acute illness in 2012 and was updated based on user feedback (as NEWS2) in December 2017 [1, 2]. Its influence extends beyond the UK, having been shown to be a useful resource in other countries (e.g. Brazil, Norway, Spain) [3–11] and found to be the most efficient of five commonly used point-based risk scores (the others being MEWS, qSOFA, BTF, SIRS) in a study spanning 28 hospitals in the USA [12]. While NEWS2 offers clinicians a useful and accessible means of identifying deterioration and triggering early intervention, and has been reported to have had a significant positive impact on patient care and safety, the continuing iterative process of healthcare improvement has been acknowledged [1] and a number of limitations have been identified. The score’s reliance on ‘normal’ physiological parameters (e.g. average vital sign ranges) when it may be argued that ‘normal’ varies by individual [13, 14] is an issue which may lead to the score suggesting a need for care escalation when not necessary [15]. Related to this, NEWS2 does not accommodate observation trends, providing a snapshot view which may miss important indications of deterioration in the individual case [15]. In a recent in-hospital study, a significant proportion of cardiac arrests were preceded by vital sign abnormalities that were not detected by NEWS2, half of which were related to increasing (or new) oxygen requirement [16]. A further limitation is that NEWS2 was designed and validated to identify patients at risk of clinical deterioration within 24 h only. It has been shown that some patients with scores indicating low 24-h risk go on to die within 30 days [17]. Collectively, these limitations suggest a need to consider modifications to NEWS2. It is now 7 years since the first formal revision of NEWS; to inform further efforts to improve the value of NEWS2, a review of relevant work since its introduction will significantly benefit the field.

**Fig. 1:**
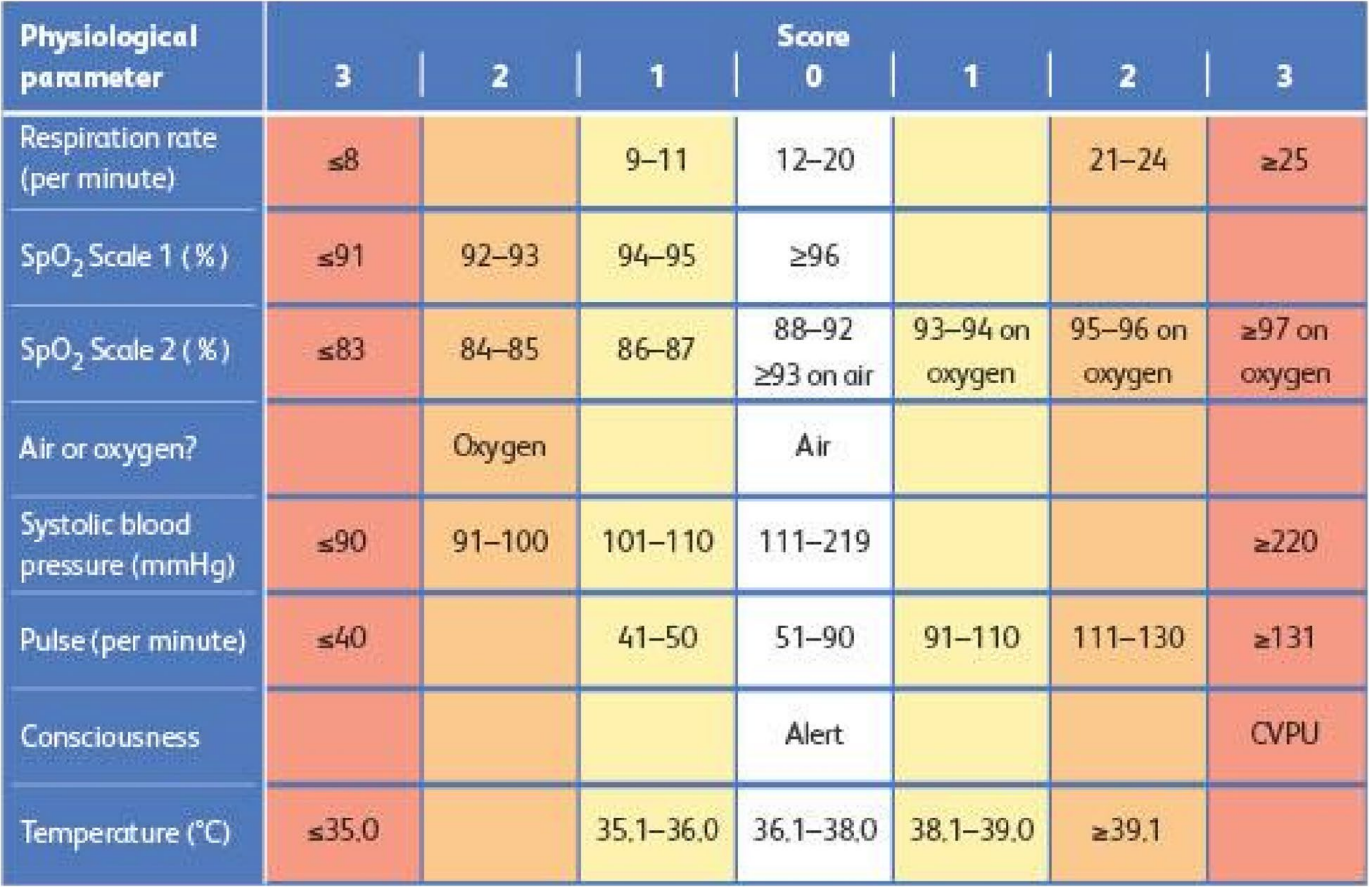
The NEWS2 scoring system [22]. *Abbreviations: mmHg: millimetres of mercury; CVPU: confusion, verbal, pain, unresponsive; °C: degrees Celsius

NEWS2 aggregates scores for six routinely collected physiological metrics: respiration rate; oxygen saturation; systolic blood pressure; pulse rate; level of consciousness or new confusion; and temperature [2] (Fig. 1). It is a generic scoring system which does not incorporate adaptations for individual characteristics or specific conditions. While this underpins its broad practical utility in clinical settings, it is also a limitation to which false alarms and reduced accuracy have been attributed [14]. For example, it has been noted that NEWS2 accuracy amongst the oldest adult group (> 85 years)—a growing and vulnerable group—needs improvement [18–20]. It has further been suggested that the standardised approach may lead to harmful correction in some cases, where elevated metrics reflect a healthy response to the illness but trigger escalated intervention as part of an early warning score trigger escalated intervention [14, 21].

A primary driver for the development of NEWS in 2012 was the recognition that a standardised approach to the detection of acute clinical deterioration would deliver substantially greater benefits overall than could be realised by the use of many approaches which may each deliver small performance advantages to specific clinical populations [1, 23]. To enhance the system, new onset confusion (in recognition of its importance in signalling decompensation) and a second oxygen saturation scale (to reduce the risk of overuse of oxygen amongst patients with hypercapnic respiratory failure) were added to NEWS to form NEWS2 which superseded the original NEWS in 2018 [1]. A key consideration in the development of NEWS was that the metrics it comprised were easily measured in the course of standard care, to enable the widespread uptake necessary to optimise the benefits offered by a standardised score, and this consideration remained when NEWS2 was developed [1]. Increasing digitisation of healthcare records and observations, together with the advancement of machine learning, are creating more ready access to, and the ability to more easily consider, a broader range of routinely collected variables which could potentially enhance NEWS2.

Reviews to date have identified needs for further investigation of how to incorporate additional metrics to extend its utility for specific illnesses [24] and how it could be enhanced by incorporating digital technologies [14, 24]. Addressing these needs, a number of publications suggest that it may be possible to strengthen the predictive accuracy of the score while placing minimal additional burden on clinicians, for example by incorporating readily available demographic data such as age [25], sex and ethnicity [26]. The literature currently lacks a review of efforts to improve the overall value and utility of NEWS2 by modifications with constant characteristics (e.g. demographics) and widely and repeatedly recorded physiological variables (e.g. vital signs); this scoping review, for which a protocol was pre-defined [27, 28], addresses this gap.

This review aimed to identify widely applicable modifications to the NEWS2 that improve its predictive accuracy without adding extra workload for clinical teams. Specifically, it focused on modifications that use constant demographic data and routinely recorded physiological variables. The influence of these modifications on NEWS2’s accuracy for adults monitored in hospital or care home settings was explored in this work.

## Methods

This review was conceived as part of a wider project investigating potential improvements to the accuracy of NEWS2 at predicting deterioration, using routinely collected demographic, observational and outcomes data from the Newcastle upon Tyne Hospitals NHS Foundation Trust. This work is outlined in Appendix A. A scoping method was selected in order to support this project in a pragmatic and timely manner.

### Scope

The Preferred Reporting Items for Systematic Reviews and Meta-Analyses Extension for Scoping Reviews (PRISMA-ScR, Appendix B) [29] was followed. The search strategy was developed using the Population, Intervention, Comparator, Outcome and Studies (PICOS) framework (Table 1) [30, 31].

**Table 1:** PICOS framework

Population	Adults of any age, demographic characteristics and clinical presentation who were monitored in hospital settings or care home settings, in any country, were included
Intervention	Studies evaluating the use of modifications or additions of other constant characteristics (e.g. demographics) or widely and repeatedly recorded physiological variables (e.g. vital signs) to the NEWS2
Comparator	No comparator required
Outcomes	The primary outcome was the types of constant characteristics and most widely and repeatedly recorded physiological variables that are being incorporated into the NEWS2 risk prediction model. Secondary outcomes included study and patient characteristics, outcome measures employed, assessments of accuracy, sensitivity and specificity, and evidence of the impact of the modifications or additions
Study types	All study types describing relevant modifications to NEWS2 were included, including protocols. Any work where no full text was available was excluded

The PICOS was adapted from the published protocol [27, 28] to focus solely on modifications or additions to NEWS2 (excluding studies relating to the now superseded NEWS). The outcomes were also defined more narrowly by specifying constant characteristics (e.g. demographics) and the most widely and repeatedly recorded physiological variables (e.g. vital signs). These refinements were implemented to improve the practical utility of the outcomes, which seek to inform potential improvements to NEWS2 that can be widely applied while placing minimal additional burden on clinical teams.

### Search strategy

The first author conducted searches of six databases between 15 and 18 April 2024: CINAHL, PubMed, Embase, ScienceDirect, Cochrane Library and Web of Science. The initial selection of keywords and MeSH terms for the searches was based on previous systematic reviews [17, 24, 26, 27, 32] and the search string was refined in collaboration with a University librarian and the second author. To ensure that all relevant papers were captured, the initial searches were designed to be broad and return all papers discussing any modification to NEWS or NEWS2, with any population, at any time point. To structure the searches, the identified keywords and MeSH terms were categorised as either NEWS or additional variables (epidemiologic factors or demography), as shown in Table 2. The full search strings and number of results returned from each database search are provided in Appendix C.

**Table 2:** Search string [27, 28]

Category	MeSH	Keywords (in title or abstract)
NEWS	Early Warning Score	“NEWS2” OR “national early warning system” OR “national early warning score” OR “track and trigger” OR “early warning score” OR “early warning system”
Additional variables	Epidemiologic Factors OR Demography	“Physiological variables” OR “physiological parameters” OR “observational data” OR “patient observation” OR “patient characteristic” OR “demographic” OR “age” OR “sex” OR “gender” OR “comorbidit*” OR “frail” OR “deprivation” OR “diastolic blood pressure” OR “urine” OR “urea” OR “oxygen therapy” OR “blood parameters” OR “physiological measures” OR “blood biomarkers” OR “ethnicity” OR “add” OR “extra” OR “supplement” OR “other factors” OR “modify” OR “modified” OR “adjusted” OR “amended”

### Eligibility criteria

The eligibility criteria for screening articles are detailed in Table 3. These criteria were adjusted from the original review protocol [27, 28] after initial screening, following further consultations with clinicians. Clinician advice that refinement was necessary to ensure clinical relevance and enhance the practical utility of the findings resulted in an eligibility criteria more focused on the objectives of the wider project the review was conducted to inform. Articles published before the introduction of NEWS2 (2018) were excluded, given the focus of this review on improvements to NEWS2. The review excluded studies involving non-universal tests (e.g. blood, urine tests, scans), in line with the objectives. Studies of paediatric populations, as well as studies not conducted within either hospital or care home settings, were also excluded to maintain relevance to NEWS2 and its intended use.

**Table 3:** Inclusion and exclusion criteria

**Inclusion criteria**	
**Protocol** [27]	**Review**
All studies that examine the modification of NEWS or NEWS2, applied to adults of any age	All studies that examine the modification of NEWS2, applied to adults of any age
Any type of additional variables or factors that could be incorporated into the standard NEWS systems	Modifications to NEWS2 by demographic or widely and repeatedly recorded epidemiologic factors (e.g. age, gender, BMI, vital signs)
Studies which discuss and justify a proposed change to the standard NEWS systems with the aim of improving accuracy or clinical impact—including by addition of a variable, simplification of the NEWS systems, or variance of the weighting of particular variables in the scoring system	As in protocol
Primary or secondary research reports, perspective articles/editorial and protocols	As in protocol
**Exclusion criteria**	
**Protocol** [27]	**Review**
Studies that focus on Track and Trigger or Early Warning Score systems that are not NEWS or NEWS2	Studies that focus on Track and Trigger or Early Warning Score systems that are not NEWS2
	Studies focussed on modifications involving non-universal tests (e.g. lactate, bilirubin, glucose, clinical frailty score)
Abstracts with no full text	As in protocol
Duplicates	As in protocol
Studies that are not published in English	As in protocol
	Studies focussed on paediatric populations
	Studies not focussed on hospital or care home settings
	Studies published before 2018

### Screening and article selection

References were exported to EndNote 21 for de-duplication and automated screening. Keyword screening was undertaken using the EndNote 21 advanced search tool (see Appendix D). Remaining references were exported to Rayyan. Further duplication of references was detected by Rayyan and resolved manually by VR.

VR and LT screened the titles and abstracts of the remaining references and discussed their decisions to arrive at an agreed set of articles for full text review. The same authors each conducted a full text review of remaining articles. LT provided clinical context to inform eligibility decisions, advising on variables that are routinely recorded across all admissions (i.e. within the review’s scope) versus specific to sub-populations or a minority of settings (i.e. out of scope.)

### Data extraction

Two reviewers (VR and LT) independently extracted data into a form, which followed the predetermined outcomes specified in the protocol paper [27, 28]. Characteristics of the study (e.g. sample size, study type, population, outcomes) and NEWS2 modifications included (number and type of modifications) were extracted. Reasons for modifications, performance of modified systems compared to that of NEWS2 and impacts on patient outcomes and clinical service delivery were also extracted where reported.

### Data analysis and synthesis

Descriptive analysis of the included references was carried out by VR and reviewed by LT. The scoping review presents a summary of this analysis, focusing on proposed and evidenced modifications to NEWS2, along with implications for future research.

## Results

### Included studies

**Fig. 2:**
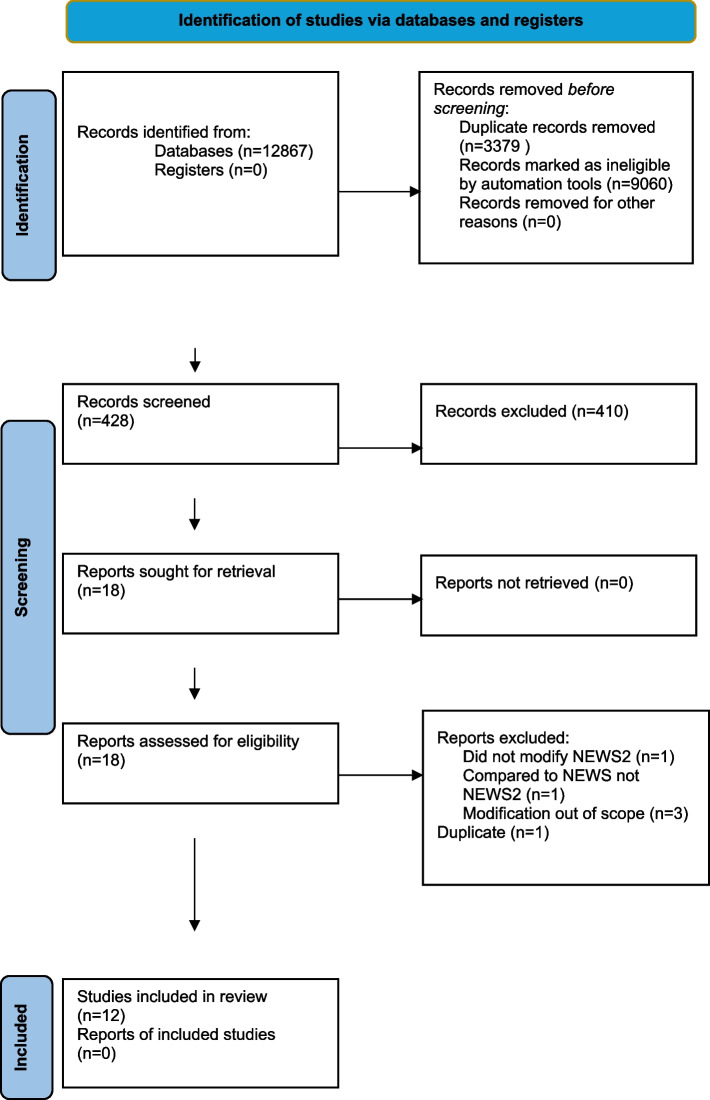
PRISMA flow diagram

A total of 12,867 references were returned by the database searches, 9488 of which remained after automated removal of duplicates by Endnote 21. Keyword searching was conducted in Endnote 21 (four passes, see Appendix D) leaving a total of 410 references for manual screening of titles and abstracts. This was conducted in Rayyan by two reviewers (VR and LT), who conferred on disagreements and ultimately determined a set of 18 references for full text review. Six references were excluded at the full text screening stage, reasons for which are detailed in the PRISMA flow diagram (Fig. 2), leaving a total of 12 included references for review.

### Study characteristics

Ten of the twelve studies employed retrospective cohorts [25, 33–41], with the other two being prospective cohort studies [42, 43]. Therefore, there was no discussion of the impact on patient outcomes or clinical service delivery (the two prospective cohort studies were not designed to evaluate these outcomes.)

Six of the studies aligned outcome measures with those for which NEWS2 was originally developed and validated by considering cardiac arrest, intensive care admission or death within 24 h, in various combinations [33–36, 40, 41], though only two employed the composite of all three validated NEWS2 outcomes [34, 40]. The other six studies employed outcome measures that deviated from those for which NEWS2 was developed, either by timeframe [25, 37, 42], clinical adverse event [39, 43] or both [38].

Study characteristics are summarised in Table 4.

**Table 4:** Included study characteristics

Author/year	Study type	Sample size	Population	Main outcome measures
Akel et al., 2021 [33]	Retrospective cohort	556,848	Medical-surgical admissions, 18 +	ICU transfer within 24 h Death within 24 h Composite of ICU transfer and death within 24 h
Clarke et al., 2023 [34]	Retrospective cohort	3704	SARS-CoV-2 / influenza patients (viral respiratory infections)	Composite of cardiac arrest, ICU admission or death within 24 h
Forster et al., 2022 [35]	Retrospective cohort	7487 (derivation) + 8739 (validation)	Respiratory patients	Death within 24 h
Gonem et al., 2022 [36]	Retrospective cohort	1100	Respiratory patients	Composite of ICU admission or death within 24 h
Kabell Nissen et al., 2022 [42]	Prospective cohort	2183	Emergency department patients, 65 +	Death within 30 days
Kamal et al., 2024 [37]	Retrospective cohort	219 (for predicting in-hospital mortality) 175 (for predicting serious illness development)	Sars-CoV-2 patients, 18 +	Serious illness within 7 days In-hospital mortality within 28 days
Malycha et al., 2019 [41]	Retrospective cohort	83,304	Hospital admissions, 18 +	In-hospital death or unplanned intensive care admission within 24 h
Maves et al., 2021 [43]	Prospective cohort	184	Hospitalised adults with confirmed COVID-19 not requiring invasive mechanical ventilation at admission	Invasive ventilation or death during entire hospital stay
Nissen et al., 2022 [25]	Retrospective cohort	14,809 (development) + 50,448 (validation)	Emergency department patients, 18 +	In-hospital mortality during entire hospital stay ICU admission during entire hospital stay
Trongtrakul et al., 2024 [38]	Retrospective cohort	725	Sars-CoV-2 patients, 18 +	Severe Covid 19 pneumonia
Yun et al., 2021 [39]	Retrospective cohort	41,687	Emergency department patients with suspected sepsis, 19 +	Septic shock within 24 h
Zhu et al., 2020 [40]	Retrospective cohort	13,319	Postoperative adult cardiac patients	Composite of cardiac arrest, ICU admission or death within 24 h

### NEWS2 modifications

Three main categories of modifications were identified: age, trend data and inspired oxygen fraction (FiO_2_). Table 5 shows the prevalence of these modification categories across the set of papers reviewed.

**Table 5:** Overview of categories of modifications

Author/year	Age	Trend data	FiO_2_	Other
Akel et al., 2021 [33]	✓			
Clarke et al., 2023 [34]			✓	
Forster et al., 2022 [35]		✓		
Gonem et al., 2022 [36]		✓		
Kabell Nissen et al., 2022 [42]				Frailty
Kamal et al., 2024 [37]	✓			
Malycha et al., 2019 [41]			✓	
Maves et al., 2021 [43]	✓			
Nissen et al., 2022 [25]	✓			
Trongtrakul et al., 2024 [38]	✓			
Yun et al., 2021 [39]	✓	✓		Gender, diastolic blood pressure
Zhu et al., 2020 [40]		✓	✓	

Ten of the twelve studies added one or more variables to NEWS2 [25, 33, 35–40, 42, 43], one of which also removed variables [33]. The other two studies investigated modifications which altered the weighting of an existing NEWS2 variable—inspired oxygen fraction (FiO_2_) [34, 41]. Six studies focused on a single modification [34, 35, 37, 42–44], three studies made 2 modifications each [25, 38, 39], one study made 4 [40] modifications, and another made 5 [33]. A further study which looked at dynamic trajectories of observations data looked at a total of 38 features [36]. In all but one study [38] (which used the C-statistic), the modifications were primarily evaluated by comparison of the area under the receiver operating characteristic curve (AUROC).

The reasons for these modifications were specific to each study and are summarised, alongside the number and types of modifications and their performance in comparison to NEWS2, in Table 6.

**Table 6:** Summary of modifications and performance compared to NEWS2

Author/ year	Number of modifications	Type of modifications	Reasons for modifications	Performance compared to NEWS2
Akel et al., 2021 [33]	5	Removed blood pressure, consciousness, temperature, oxygen data and added age	To limit the number of variables by focusing on the most important predictors, in order to widen usage and applicability	Better at predicting ICU transfer within 24 h and ICU transfer combined with death within 24 h Worse at predicting death within 24 h alone
Clarke et al., 2023 [34]	1	Changed inspired oxygen fraction from binary to weighted categorical variable	Patient deterioration is associated with increasing oxygen requirements; NEWS2 may be improved by providing additional weight as oxygen requirement increases	Better at predicting cardiac arrest, transfer to ICU or death within 24 h
Forster et al., 2022 [35]	1	Maximum NEWS2 score in preceding 24 h	To improve predictive accuracy using a simple metric capable of being widely adopted	Better at predicting death within 24 h
Gonem et al., 2022 [36]	Not available	Trends and variability in clinical observations	To take into account greater detail and time series data	Better at predicting a composite of ICU admission or death within 24 h
Kabell Nissen et al., 2022 [42]	1	Clinical frailty score	To improve risk stratification for older people as approaches based on vital signs lack accuracy to predict death	Better at predicting death within 30 days
Kamal et al., 2024 [37]	1	Age	Not reported—this study did not develop the modification	Better at predicting serious illness within 7 days Worse at predicting in-hospital mortality within 28 days
Malycha et al., 2019 [41]	1	Inspired oxygen fraction as a weighted categorical variable	To determine if scoring FiO_2_ in NEWS improved performance when predicting in-hospital death and unplanned ICU admission	Better at predicting in-hospital death and unplanned ICU admission within 24 h
Maves et al., 2021 [43]	1	Age	Evidence is mixed relating to the predictive accuracy of adding age to NEWS2 for Sars-CoV-2 patients	Similar at predicting invasive ventilation or death
Nissen et al., 2022 [25]	1	Age	To evaluate if the addition of age improved the predictive accuracy of NEWS2	Better at predicting in-hospital mortality Similar at predicting ICU admission
Trongtrakul et al., 2024 [38]	2	Age BMI	Sought to improve NEWS2 predictive accuracy for severe Covid 19 pneumonia and had identified age and BMI as associated with the outcome	Better at predicting severe Covid 19 pneumonia
Yun et al., 2021 [39]	8	Age Gender Diastolic blood pressure Plus 5 vital signs	To improve NEWS2 prediction of septic shock using readily available data	Better at predicting septic shock within 24 h
Zhu et al., 2020 [40]	4	Increased oxygen therapy categories from 2 to 4 Vital sign trends	Patients who are stable or improving but meet NEWS2 escalation thresholds contribute to a high ‘non-event’ rate which erodes confidence in NEWS2	Better at predicting a composite of cardiac arrest, ICU admission or death within 24 h

#### Addition of ‘age’

Six studies added an age variable to NEWS2 [25, 33, 37–39, 43]. Age was the only additional variable considered in three studies, and its impact on the predictive accuracy of NEWS2 was mixed. One study found the addition of age improved the accuracy of NEWS2 at predicting the development of serious illness within 7 days of hospital admission but was less accurate than the standard NEWS2 at predicting in-hospital mortality within 28 days of admission [37]. Another found that age enhanced the accuracy of NEWS2 for predicting in-hospital mortality but not for predicting admission to intensive care [25]. The third study looking exclusively at age found the addition of age to perform similarly to NEWS2 at predicting invasive ventilation and death [43].

Three studies incorporated age as a variable alongside other modifications. A simplified model which retained only heart rate and respiratory data from NEWS2 and also added age performed significantly better than NEWS2 at predicting transfer to intensive care and a combined outcome of transfer to intensive care or death within 24 h but did not perform as well as NEWS2 at predicting death alone [33]. This study noted that respiratory rate and heart rate were two of the strongest predictors across all the scores they evaluated. A study which added age and body mass index (BMI) to NEWS2 found that each additional variable separately improved discriminative ability to predict severe COVID 19 pneumonia amongst SARS-CoV-2 patients (BMI only moderately) and the best improvement was observed when age and BMI were both added to NEWS2 [38]. The final study which incorporated age developed an algorithm which included NEWS2 plus age, gender and six vital signs [39]. It outperformed the model based only on NEWS2 for prediction of septic shock.

#### Addition of trend data

Three of the studies examined the impact of adding trend data to NEWS2 [35, 36, 40]. The first of these added maximum NEWS2 score in the preceding 24 h to NEWS2, which moderately improved the accuracy of prediction of death within 24 h over NEWS2 alone [35]. One included 38 time series features in total, including variance from the previous observation, average and standard deviation of the 3–5 previous observations and a range of categorisations of previous observations ranging from normal and stable to outside normal range and worsening [36]. Its performance was better than that of NEWS2 comparing the composite outcome measures of death, intensive care admission within 24 h, and clinically significant deterioration within 4 h. The final study developed a model which included the most recent rate of change of vital signs and their level and variability across the three previous observations and all sequential values [40]. It also added two additional oxygen therapy categories and included frequency of observations over the previous 6 h. This model performed better than NEWS2 at predicting a composite of cardiac arrest, unplanned intensive care admission or death within 24 h, amongst post-operative cardiac patients.

#### Other variables incorporated into NEWS2

Three studies modified the incorporation of inspired oxygen fraction (FiO2) within NEWS2 by changing it from a binary component to a weighted variable [34, 40, 41]. The two which looked exclusively at FiO_2_ [34, 41] both reported improvements to the performance of NEWS2 for the composite outcomes of peri-arrest, cardiac arrest, unplanned critical care and in hospital death or unplanned ICU admission respectively. The other paper in this group incorporated an FiO_2_ weighting change with the addition of trend data and also reported an improvement for the composite outcome of cardiac arrest, unplanned ICU admission or death within 24 h [40].

The final study focused on emergency department patients over 65 added the clinical frailty scale (CFS) to NEWS2 to predict death within 30 days [42]. It found the AUROC of the modified score to be significantly higher than that of NEWS2.

## Discussion

### Summary of findings

Three of the studies reviewed focused on FiO_2_—increasing detail and weighting—and reported significant improvements to NEWS2, therefore identifying a variable meriting further investigation [34, 40, 41]. Positive results were reported from all three studies which added trend data to the NEWS2 score [35, 36, 40], suggesting that the snapshot nature of NEWS2 may limit its predictive accuracy [15]. The mixed results found when age was added to NEWS2 suggest that further research is required to determine whether modifications for older people would improve its accuracy [18].

These findings indicate that it may be possible to improve the predictive accuracy of NEWS2 by the addition of widely available demographic data and/or trends in routinely recorded physiological measurements.

### Strengths and weaknesses of studies

The studies collectively adopted a range of outcomes, only half of which were consistent with the outcomes for which NEWS2 was developed and validated. Deviation from NEWS2 outcomes, in the absence of validation of the established score, weakens the case for comparing the performance of NEWS2 to modified versions and raises the concern that the modifications were tested against typical NEWS2 outcomes, without these results being reported. However, there is a strong rationale for investigating the utility of NEWS2 and any modifications in predicting alternative outcomes, as this may produce clinically valuable findings. This work may benefit from analysis of both standard NEWS2 outcomes and novel outcome measures.

The review process found that a small number of studies published after the introduction of NEWS2 state that they investigated modifications or made comparisons to the original NEWS [33, 42]. As NEWS2 superseded NEWS in the UK in 2018, this makes it difficult to be certain which version of the score was used and highlights the importance of clarifying this in future publications.

### Strengths and limitations of review

This review is focused on modifications to NEWS2 which could be widely applicable in clinical practice without placing an additional burden on clinical teams, in order to direct future research towards strengthening the utility and value of NEWS2 at scale. Its relatively narrow and disciplined focus means that some informative studies, with promising results, were excluded. For example, a study evaluating the addition of a patient wellness score was excluded because it was completed in 2017 and therefore used NEWS rather than NEWS2 [45]. Similarly, as this review was limited to studies which made modifications to NEWS2 by addition, subtraction or weighting changes to variables, studies which reported the results of machine learning or algorithm studies which did not include in-scope modifications were also excluded.

Adherence to the early stages of a predetermined protocol [27, 28] added strength to the study as it ensured a structured, comprehensive literature search. Deviation from the original protocol (in response to additional input from clinical colleagues) beyond the automated screening stage is a further strength of the review; this flexible approach enabled the team to refine the scope of the study to ensure its practical utility. While this deviation from the protocol meant that some of the search terms initially employed were unnecessary, resulting in a larger initial reference set than ultimately needed, their inclusion did not detract from the final output. As the original protocol was made available by publication, it was not registered with Open Science Framework which has been identified as a limitation.

A recognised limitation is that of positive publication bias, which may be a particular risk for modelling research in which cohort data on a wide range of variables is available; researchers are able to ‘play with the data’ until a positive effect is identified and report selectively. Linked to this is the limitation that, as a scoping review, no analysis of the quality of studies reviewed is included. An earlier review of the methodologies adopted in developing early warning scores found issues in most—and risk of bias in all—included studies [46]; our findings, therefore, must be interpreted with appropriate caution. Partially mitigating these considerations is the fact that the search was restricted to peer-reviewed sources, though while adding a degree of reassurance, this also means that relevant insights from other sources may have been missed.

The papers included in the review exhibit a number of sources of clinical heterogeneity, with differing populations, sample sizes, outcome measures and study designs. The review makes these variables explicit in its summary of the literature and the differences should be kept in mind when reading the synthesis provided.

### Findings in relation to recent literature

A 2023 evaluation found that NEWS2 either outperformed or matched the performance of 36 other early warning scores in 120 out of 123 patient groups [47], supporting its continued, broad usage and the value of ongoing research to improve its utility. It has been suggested that further improvements to the predictive accuracy of NEWS2 for 24-h mortality are unlikely to add substantial clinical value. Instead, further efforts should be directed towards alternative enhancements, such as improving the system’s ease of use, efficiency or associated health outcomes [14]. This argument supports the rationale of those studies which sought to simplify NEWS2, tested machine learning approaches and adopted outcome measures which deviated from those for which NEWS2 is already well-validated. The same authors highlight that use of NEWS2, as a universal score, risks suboptimal effectiveness in various circumstances (e.g. when small increases in vital sign metrics are a helpful response to the illness and when a patient’s normal parameters are outside the average range) and that omitting potentially feasible metrics could lead to missing certain conditions (e.g. urine output for acute kidney injury, diastolic blood pressure for early distributive shock) [14]. In focusing on additional metrics that do not place additional burden on clinical teams, our review only considered studies that maintained the ease of use of NEWS2 while potentially improving overall predictive accuracy, often by taking individual characteristics which impact NEWS2 variables into account (e.g. age).

A recent meta-analysis found that NEWS2 demonstrated high sensitivity and specificity in predicting 2-day mortality but poor predictive accuracy for death during the entire hospital stay and within 30 days [32]. In line with our rationale and findings, validation for 2-day outcomes confirms the value of NEWS2, but its poor long-term performance indicates a need for further improvements.

### Future research

Promising performance in predicting one of the main NEWS2 outcomes with a version which retained only 3 NEWS2 variables suggests that further efforts to maintain predictive accuracy with simplified modifications of the score may be warranted. Such variations could offer rapid and easily implemented means to identify the risk of deterioration in a wide range of settings. Future research may investigate combinations of selected NEWS2 variables plus 1–2 additional variables, for example.

Age has received attention as a universally available potential predictor of risk of clinical decline, with investigations to date reporting mixed findings. Within this review, studies of the impact of modifying NEWS2 with an age variable adopted heterogeneous outcome measures, which may be affected differently by age. In addition to age itself affecting patient outcomes, it may be associated with variable clinical approaches and treatment options that complicate efforts to understand its role in predicting patient deterioration [48]. Given the substantial potential but lack of clarity we have found, this area of research would benefit at this stage from further studies designed to explore the complex effects of age on the specific outcomes for which NEWS2 is well validated (ICU transfer or death within 24 h), before expanding to others in due course.

Studies which reported modifications based on trends in the NEWS2 score are another area of focus. Increasingly widespread digital recording of patient data and rapid advances in machine learning mean that algorithms may be developed to interrogate large datasets and identify trends and variations which may enhance the predictive power of NEWS2. It is recommended that such work should be aligned to the outcome measures validated for NEWS2 in order to ensure like-for-like comparisons before testing the algorithms’ predictive accuracy for other outcomes or time horizons. Comprehensive reporting of all variables and outcomes tested and results is necessary in all future work of this nature. Our subsequent study will explore how additional variables or new weightings could improve NEWS2’s accuracy in predicting patient deterioration and develop a proof-of-concept model using data from an NHS trust. Collaboration across groups with synergistic interests is strongly encouraged to facilitate knowledge sharing and drive system improvements. To this end, an open invitation to collaborate and the working parameters of the Newcastle upon Tyne Hospitals NHS Foundation Trust dataset, which this review informed, are included in Appendix A.

Another gap in literature identified from this review is related to the lack of understanding about algorithm-driven enhancement of NEWS2. Specifically, while some machine learning or algorithm studies were included in this review, those which did not include the types of modifications within the scope of this review were excluded. There is currently no published, structured review of machine learning or algorithm studies seeking to improve the predictive accuracy of NEWS2, so the authors are conducting a systematic review of such work to contribute further to the field.

## Conclusions

This review has identified three key types of modifications that may improve the predictive accuracy of NEWS2: addition of age, more nuanced treatment of FiO_2_ data and incorporation of observational trend data. These modifications align with the original objective of the review, focusing on broadly applicable and widely recorded variables that do not place additional burden on clinicians. While further evidence in all cases is needed, our review concludes that future research should also investigate the composite impact of age, FiO_2_ and trend data modifications to NEWS2. Collectively, these promising modifications have the potential to drive substantial improvements with broad utility.

## Data Availability

No datasets were generated or analysed during the current study.

## Appendix A

### Collaboration invitation and dataset parameters

#### Improving the ability of the National Early Warning Score (NEWS2) system to predict critical outcomes through additional patient data or amendments to the scoring process

Executive summary: Our research group is investigating potential improvements to the National Early Warning Score (NEWS2). A number of parties working with similar objectives have expressed interest in collaborating, which is anticipated to take the form of opportunities to test developed algorithms on others’ datasets. This document provides next steps to establish collaboration and the Newcastle upon Tyne Hospitals NHS Foundation Trust working dataset parameters for reference.

Context: NEWS2 has been adopted as the standard approach for early detection of deterioration in clinical settings in the UK. Despite its widespread use, limitations have been identified with the score, including a reliance on 'normal' physiological parameters without accounting for individual variation (e.g. age), and a focus on predicting deterioration within 24 h.

This project will use routinely collected demographic, observational and outcomes data from the Newcastle upon Tyne Hospitals NHS Foundation Trust to examine how additional variables and/or new weightings could improve the accuracy of NEWS2 at predicting deterioration and develop and test a proof-of-concept model. Refining the NEWS2 system to improve its accuracy, particularly for older adults, will increase its clinical value in the long term, as the population ages.

A scoping review has provided an overview of modifications to the National Early Warning Score 2 in recent literature and their impact on the accuracy of the score, to inform this data extraction protocol. In brief, the review identified the addition of age and vital signs trend data, along with the transformation of inspired oxygen fraction (FiO2) from a binary to a weighted variable, as showing promise for improving the predictive accuracy of NEWS2.

Our working parameters for our dataset are outlined over the following pages.

Next steps: We are currently exporting Electronic Health Records data in preparation for initial algorithm development. We invite NHS Trusts and non-UK health service providers to collaborate with us on this important project in the following ways:

1. Discuss a test of algorithms you have developed, aligned with our objectives, on our dataset for validation and refinement
2. Discuss a test of our algorithm on your dataset
3. Discuss any other knowledge-sharing or synergy-seeking suggestions

Please contact cen.cong@newcastle.ac.uk to progress.

We look forward to working together.

### Dataset parameters

#### Cohort

- Admitted in-patients with recorded electronic observations between (start date to be confirmed) and (end date to be confirmed)
- Patients attending the Emergency Department with recorded electronic observations between (start date to be confirmed) and (end date to be confirmed) and who were admitted as an inpatient from the ED attendance

#### Exclusions

- National opt-out patients
- ED attendances which did not result in an inpatient admission
- Observations not recorded using NEWS2: patients aged under 16; patients aged 16–18 treated on a paediatric ward; maternity patients; critical care patients

#### Included fields

**Table:**

**Dataset**	**Field**	**Data type**	**Notes**
Encounters	Patient ID	Numeric	To be generated through pseudonymisation of NHS Number
	Encounter ID	Numeric	Identifier for the hospital admission encounter
	Encounter type	Character	Inpatient / Emergency Department
	Age	Numeric	On admission
	Sex	Character	Biological Sex
	Ethnicity	Character	Categories as defined in the NHS Data Dictionary (https://www.datadictionary.nhs.uk/data_elements/ethnic_category.html)
	Death	Character	Flag field to indicate whether the patient outcome of the hospital encounter was death
	Cardiac arrest	Character	Flag field to indicate whether the patient was coded as with cardiac arrest during the hospital encounter
	Resuscitation	Character	Flag field to indicate whether the patient was coded as needing resuscitation during the hospital encounter (noting this would be any level of resus above basic CPR)
	Unplanned admission to critical care	Character	Flag field to indicate whether the patient had an unplanned admission to critical care during the hospital encounter
BMI	Encounter ID	Numeric	To link to Encounters dataset
	Height	Numeric	All instances recorded against the encounter
	Height units	Character	
	Height date time	DateTime	
	Weight	Numeric	
	Weight units	Character	
	Weight date time	DateTime	
	BMI	Numeric	
	BMI date time	DateTime	
Observations	Encounter ID	Numeric	To link to Encounters dataset
	Heart Rate	Numeric	NEWS2 algorithm variable
	Respiratory Rate	Numeric	
	Systolic Blood Pressure	Numeric	
	Oxygen Saturation	Numeric	
	Oxygen Therapy	Numeric	
	Temperature	Numeric	
	AVPU	Character	
	Diastolic Blood Pressure	Numeric	Additional variable within NuTH e-obs set
	Nursing Concern	Character	
	Urine Output	Character	
	Pain Score	Character	
	Mask Code	Character	
	Oxygen Therapy Percentage	Character	
	Paired Value One (lying blood pressure)	Character	
	Paired Value Two (standing blood pressure)	Character	
	Observation date time	DateTime	
	NEWS2 score	Numeric	
	NuTH Risk Rating	Numeric	Risk rating generated from the observation set within the eObs system
	Partial Obs Set (NEWS2 variables)	Character	Flag to identify if the observation set was complete or partial in respect of the NEWS2 algorithm variables
	Partial Obs Set (Additional variables)	Character	Flag to identify if the observation set was complete or partial in respect of additional NuTH e-obs variables
	Observation location	Character	Ward location where the observation set was recorded

## Appendix B

### Preferred Reporting Items for Systematic Reviews and Meta-Analyses extension for Scoping Reviews (PRISMA-ScR) Checklist

**Table:**

**Section**	**Item**	**PRISMA-ScR Checklist item**	**Reported in section**
**TITLE**			
Title	1	Identify the report as a scoping review.	Front page
**Abstract**			
Structured summary	2	Provide a structured summary that includes (as applicable): background, objectives, eligibility criteria, sources of evidence, charting methods, results, and conclusions that relate to the review questions and objectives.	Abstract
**Introduction**			
Rationale	3	Describe the rationale for the review in the context of what is already known. Explain why the review questions/objectives lend themselves to a scoping review approach.	Abstract; Background
Objectives	4	Provide an explicit statement of the questions and objectives being addressed with reference to their key elements (e.g. population or participants, concepts, and context) or other relevant key elements used to conceptualise the review questions and/or objectives.	Abstract; Background
**Methods**			
Protocol and registration	5	Indicate whether a review protocol exists; state if and where it can be accessed (e.g. a Web address); and if available, provide registration information, including the registration number.	Methods - Scope
Eligibility criteria	6	Specify characteristics of the sources of evidence used as eligibility criteria (e.g. years considered, language, and publication status), and provide a rationale.	Methods - Eligibility criteria
Information sources^a^	7	Describe all information sources in the search (e.g. databases with dates of coverage and contact with authors to identify additional sources), as well as the date the most recent search was executed.	Methods - Search strategy
Search	8	Present the full electronic search strategy for at least 1 database, including any limits used, such that it could be repeated.	6 Appendix C
Selection of sources of evidence^b^	9	State the process for selecting sources of evidence (i.e., screening and eligibility) included in the scoping review.	Methods - Screening and article selection
Data charting process^c^	10	Describe the methods of charting data from the included sources of evidence (e.g. calibrated forms or forms that have been tested by the team before their use, and whether data charting was done independently or in duplicate) and any processes for obtaining and confirming data from investigators.	N/A
Data items	11	List and define all variables for which data were sought and any assumptions and simplifications made.	Methods - Search strategy
Critical appraisal of individual sources of evidence^d^	12	If done, provide a rationale for conducting a critical appraisal of included sources of evidence; describe the methods used and how this information was used in any data synthesis (if appropriate).	N/A
Synthesis of results	13	Describe the methods of handling and summarising the data that were charted.	Methods - Data extraction; Data analysis and synthesis
**Results**			
Selection of sources of evidence	14	Give numbers of sources of evidence screened, assessed for eligibility, and included in the review, with reasons for exclusions at each stage, ideally using a flow diagram.	Results - Included studies
Characteristics of sources of evidence	15	For each source of evidence, present characteristics for which data were charted and provide the citations.	Results - Study characteristics
Critical appraisal within sources of evidence	16	If done, present data on critical appraisal of included sources of evidence (see item 12).	N/A
Results of individual sources of evidence	17	For each included source of evidence, present the relevant data that were charted that relate to the review questions and objectives.	Results - Study characteristics
Synthesis of results	18	Summarise and/or present the charting results as they relate to the review questions and objectives.	Results - NEWS2 modifications
**Discussion**			
Summary of evidence	19	Summarise the main results (including an overview of concepts, themes, and types of evidence available), link to the review questions and objectives, and consider the relevance to key groups.	Discussion - Summary of findings; Strengths and weaknesses of studies
Limitations	20	Discuss the limitations of the scoping review process.	Discussion - Strengths and limitations of review
Conclusions	21	Provide a general interpretation of the results with respect to the review questions and objectives, as well as potential implications and/or next steps.	Conclusions
**Funding**			
Funding	22	Describe sources of funding for the included sources of evidence, as well as sources of funding for the scoping review. Describe the role of the funders of the scoping review.	Funding19

*JBI* Joanna Briggs Institute, *PRISMA-ScR* Preferred Reporting Items for Systematic reviews and Meta-Analyses extension for Scoping Reviews

^a^Where *sources of evidence* (see second footnote) are compiled from, such as bibliographic databases, social media platforms, and Web sites

^b^A more inclusive/heterogeneous term used to account for the different types of evidence or data sources (e.g. quantitative and/or qualitative research, expert opinion, and policy documents) that may be eligible in a scoping review as opposed to only studies. This is not to be confused with *information sources* (see first footnote)

^c^The frameworks by Arksey and O’Malley (6) and Levac and colleagues (7) and the JBI guidance (4, 5) refer to the process of data extraction in a scoping review as data charting*.*

^d^The process of systematically examining research evidence to assess its validity, results, and relevance before using it to inform a decision. This term is used for items 12 and 19 instead of "risk of bias" (which is more applicable to systematic reviews of interventions) to include and acknowledge the various sources of evidence that may be used in a scoping review (e.g. quantitative and/or qualitative research, expert opinion, and policy document)

## Appendix C

### Sample search strings

**Table:**

**Database**	**Search string**	**Results**
PubMed, Searched April 17	((Early Warning Score[MeSH Terms]) OR ("NEWS2" OR "national early warning system" OR "national early warning score" OR "track and trigger" OR "early warning score" OR "early warning system")) AND ((Epidemiologic Factors OR Demography[MeSH Terms]) OR ("Physiological variables" OR "physiological parameters" OR "observational data" OR "patient observation" OR "patient characteristic" OR "demographic" OR "age" OR "sex" OR "gender" OR "comorbidit" OR "frail" OR "deprivation" OR "diastolic blood pressure" OR "urine" OR "urea" OR "oxygen therapy" OR "blood parameters" OR "physiological measures" OR "blood biomarkers" OR "ethnicity" OR "add" OR "extra" OR "supplement" OR "other factors" OR "modify" OR "modified" OR "adjusted" OR "amended"))	*1856*
Embase<1974 to 2024 April 16> Searched April 17	((early warning score/) or ("NEWS2" or "national early warning system" or "national early warning score" or "track and trigger" or "early warning score" or "early warning system").ti,ab,kw.) AND ((epidemiology/ or demography/) or ("Physiological variables" or "physiological parameters" or "observational data" or "patient observation" or "patient characteristic" or "demographic" or "age" or "sex" or "gender" or "comorbidit" or "frail" or "deprivation" or "diastolic blood pressure" or "urine" or "urea" or "oxygen therapy" or "blood parameters" or "physiological measures" or "blood biomarkers" or "ethnicity" or "add" or "extra" or "supplement" or "other factors" or "modify" or "modified" or "adjusted" or "amended").ti,ab,kw.)	*2321*
ScienceDirect, searched April 17	("early warning score" OR "early warning system" OR "NEWS2") AND ("Epidemiologic factors" OR "Physiological variables" OR "demographic" OR "age" OR "gender" OR "modif!")	*9982 -> Can only export a maximum of* ***6000.*** *Therefore limited to English only, and selected “research articles.” The search says ordered by relevance, so the first 6000 of the remaining 6107 results were exported*
CINAHL, searched April 15	((MM "Early Warning Score") or "NEWS2" OR "national early warning system" OR "national early warning score" OR "track and trigger" OR "early warning score" OR "early warning system") AND ("Epidemiologic factors" OR "demography" OR "Physiological variables" OR "physiological parameters" OR "observational data" OR "patient observation" OR "patient characteristic" OR "demographic" OR "age" OR "sex" OR "gender" OR "comorbidit" OR "frail" OR "deprivation" OR "diastolic blood pressure" OR "urine" OR "urea" OR "oxygen therapy" OR "blood parameters" OR "physiological measures" OR "blood biomarkers" OR "ethnicity" OR "add" OR "extra" OR "supplement" OR "other factors" OR "modify" OR "modified" OR "adjusted" OR "amended")	*691*
Cochrane Library, searched April 17	(MeSH descriptor: [Early Warning Score] explode all trees OR "NEWS2" OR "national early warning system" OR "national early warning score" OR "track and trigger" OR "early warning score" OR "early warning system") AND (MeSH descriptor: [Epidemiologic Factors] explode all trees OR MeSH descriptor: [Demography] explode all trees OR "Physiological variables" OR "physiological parameters" OR "observational data" OR "patient observation" OR "patient characteristic" OR "demographic" OR "age" OR "sex" OR "gender" OR "comorbidit" OR "frail" OR "deprivation" OR "diastolic blood pressure" OR "urine" OR "urea" OR "oxygen therapy" OR "blood parameters" OR "physiological measures" OR "blood biomarkers" OR "ethnicity" OR "add" OR "extra" OR "supplement" OR "other factors" OR "modify" OR "modified" OR "adjusted" OR "amended"	*211*
Web of Science, searched April 18	ALL=(((early warning score) or ("NEWS2" or "national early warning system" or "national early warning score" or "track and trigger" or "early warning score" or "early warning system").ti,ab,kw.) AND ((epidemiology or demography) or ("Physiological variables" or "physiological parameters" or "observational data" or "patient observation" or "patient characteristic" or "demographic" or "age" or "sex" or "gender" or "comorbidit" or "frail" or "deprivation" or "diastolic blood pressure" or "urine" or "urea" or "oxygen therapy" or "blood parameters" or "physiological measures" or "blood biomarkers" or "ethnicity" or "add" or "extra" or "supplement" or "other factors" or "modify" or "modified" or "adjusted" or "amended")))	*1788*
Total		*12867*
Total after duplicates removed		*9488*

## Appendix D

### EndNote screening

**Table:**

**Pass**	**Search string**	**# of references remaining**	**Test articles present?**
1	ANY FIELD: national early warning	982	yes
2^a^	ABSTRACT: Epidemiologic OR demograph OR physiologic OR observation OR patient characteristic OR blood OR ethnic OR age OR sex OR gender	901 Pass 2: 812 Pass 3: 586	yes
3^a^	ABSTRACT: frail OR deprivation OR comorbid OR urine OR urea OR modif OR adjust OR amend OR supplement OR add		yes
4	ABSTRACT: news AND (hospital OR home OR setting) AND (modif OR add OR supplement OR amend OR adjust)	428	yes

^a^Endnote limits screening to 10 terms so passes 2 and 3 were combined, duplicates removed

## Abbreviations

AUROC: Area under the receiving operator curve
BTF: Between the Flags
CFS: Clinical Frailty Scale
CVPU: New confusion, voice, pain, unresponsive
FiO2: Fraction of inspired oxygen
ICU: Intensive care unit
MEWS: Modified Early Warning Score
mmHg: Millimetres of mercury
NEWS: National Early Warning Score
PICOS: Population, Intervention, Comparator, Outcome and Studies
PRISMA-ScR: Preferred Reporting Items for Systematic Reviews and Meta-Analyses Extension for Scoping Reviews
qSOFA: Quick sequential organ failure assessment
Sars-Cov-2: Severe acute respiratory syndrome coronavirus 2
SIRS: Systemic inflammatory response syndrome
